# Investigation of factors influencing low physical activity levels in community-dwelling older adults with chronic pain: a cross-sectional study

**DOI:** 10.1038/s41598-023-41319-7

**Published:** 2023-08-28

**Authors:** Mitsumasa Hida, Ryota Imai, Misa Nakamura, Hidetoshi Nakao, Kodai Kitagawa, Chikamune Wada, Shinji Eto, Masatoshi Takeda, Masakazu Imaoka

**Affiliations:** 1 Department of Rehabilitation, Osaka Kawasaki Rehabilitation University, 158 Mizuma, Kaizuka, Osaka 597-0104 Japan; 2 Department of Physical Therapy, Josai International University, 1 Gumyo, Togane, Chiba 283-8555 Japan; 3 National Institute of Technology, Hachinohe College, 16-1 Uwanotai, Tamonoki, Hachinohe, Aomori 039-1192 Japan; 4 Graduate School of Life Science and Systems Engineering, Kyushu Institute of Technology, Hibikino 2-4, Wakamatsu-ku, Kitakyushu, Fukuoka 808-0135 Japan

**Keywords:** Health care, Rheumatology

## Abstract

Low levels of physical activity in individuals with chronic pain can lead to additional functional impairment and disability. This study aims to investigate the predictors of low physical activity levels in individuals with chronic pain, and to determine the accuracy of the artificial neural network used to analyze these predictors. Community-dwelling older adults with chronic pain (n = 103) were surveyed for their physical activity levels and classified into low, moderate, or high physical activity level groups. Chronic pain-related measurements, physical function assessment, and clinical history, which all influence physical activity, were also taken at the same time. Logistic regression analysis and analysis of multilayer perceptron, an artificial neural network algorithm, were performed. Both analyses revealed that history of falls was a predictor of low levels of physical activity in community-dwelling older adults. Multilayer perceptron analysis was shown to have excellent accuracy. Our results emphasize the importance of fall prevention in improving the physical activity levels of community-dwelling older adults with chronic pain. Future cross-sectional studies should compare multiple analysis methods to show results with improved accuracy.

## Introduction

Chronic pain (CP) is present in 1.5 billion people worldwide and is a public health problem^1^. CP can cause negative psychological effects and can lead to low activity, depression, and reduced activities of daily living^2, 3^. The reduced physical activity levels of individuals with CP can lead to further functional impairment and disability, so there have been various recommended exercise programs to increase physical activity^4, 5^. Despite the perceived benefits of increasing physical activity levels for CP, the causes of low physical activity levels in individuals with CP are not well understood. The limited available research has reported that factors contributing to low physical activity levels in individuals with CP are complex and varied. Qualitative research suggests that a variety of factors influence barriers to physical activity in adults with CP, including pain, fatigue, and risks associated with physical activity^6^. Kinesiophobia and pain tolerance thresholds also influence low physical activity levels in adults with CP^7, 8^. However, all previous studies have compared CP groups with healthy controls. There has not been a comparison of individuals with CP with different levels of physical activity.

Treating and resolving CP itself is fundamentally difficult, so the American Society of Anesthesiologists Task Force on Chronic Pain Management and the American Society of Regional Anesthesia and Pain Medicine have identified goals for the treatment of patients with CP. These include pain management, functional capacity, improved physical and mental health, and improved quality of life^9^. The American Society of Regional Anesthesia and Pain Medicine lists goals of treatment for CP as improving pain management, improving functional capacity, improving physical and mental health, and improving quality of life. Deficits in regular physical activity have a variety of negative effects on CP, so research must focus upon groups of patients with CP with low levels of physical activity^7, 10, 11^.

This study aims to extract factors that predict low levels of physical activity among community-dwelling elderly people with CP. We used multilayer perceptron (MLP), an artificial neural network (ANN), to extract the characteristics of individuals with low physical activity. Prediction and feature importance using ANN is a form of analysis that has been notably used in COVID-19 research, and in analysis of factors related to severity of illness, including in predicting severity of illness at an early stage^12, 13^. ANN has been applied in classification, prediction, feature selection, and feature importance. We therefore considered that the functions of prediction and feature importance extraction by ANN would be ideal for the purpose of this study. Machine learning analysis has not yet been used in survey research on CP populations, but it could provide a better predictive model. A growing number of studies have demonstrated that ANN have better predictive accuracy than traditional regression analysis^14, 15^. This study seeks to validate the accuracy of using MLP as a model for predicting physical activity levels among community-dwelling older adults with CP, and to compare it with a conventionally used logistic regression (LR) model.

## Methods

This is a cross-sectional analysis of data related to health checks conducted in cooperation between Osaka Kawasaki Rehabilitation University and Kaizuka City. Health checks were conducted at three community centers in Kaizuka City, Osaka, Japan, between 2021 and 2022. This study is reported following the STROBE guidelines^16^.

### Participants

The inclusion criteria for this study were as follows: (1) age ≥ 65 years; (2) living independently at home; (3) no cardiac pacemaker; and (4) presence of chronic pain. Chronic pain defined by the International Association for the Study of Pain: “Chronic pain is pain that persists or recurs for longer than 3 months”. Individuals who could not answer the health check questions due to visual, hearing, or cognitive impairments were excluded from the study.

### Ethical consideration

All participants provided written, informed consent to inclusion in the research study. This study was approved by the Osaka Kawasaki Rehabilitation University Ethics Committee (reference number OKRU30-A016) and was conducted in accordance with the Declaration of Helsinki.

### Demographic data

Subjects completed a questionnaire on their age, gender, height, and weight. Body mass index (BMI) was calculated by dividing their body weight (kg) by their height squared (m^2^).

### Daily activity assessment

International Physical Activity Questionnaire Environmental Module Short Form (IPAQ-SF) was used to assess the degree of physical activity of the study participants^17^. It asks about three specific types of activity in four domains (leisure, work, household activities, and transportation) that took place in the previous seven days. The items are structured to provide separate scores for walking, moderately strenuous activity, and very strenuous activity. Respondents were categorized into one of three groups according to their physical activity level.

High Physical Activity Level (HPAL) was defined in IPAQ-SF as any of the following:

- Vigorous-intensity activity on at least three days, achieving a minimum total physical activity of at least 1500 MET-minutes/week.
- Seven or more days of any combination of walking, moderate-intensity or vigorous-intensity activity, achieving a minimum total physical activity of at least 1500 MET-minutes/week.
- Seven or more days of any combination of walking, moderate-intensity or vigorous-intensity activities, achieving a minimum total physical activity of at least 3000 MET-minutes/week.

Moderate Physical Activity Level (MPAL) was defined in IPAQ-SF as any of the following:

- Three or more days of vigorous-intensity activity of at least 20 min per day.
- Five or more days of moderate-intensity activity.
- Five or more days of any combination of walking, moderate-intensity or vigorous-intensity activities, achieving a minimum total physical activity of at least 600 MET-minutes per day.
- Five or more days of moderate-intensity activity and/or walking at least 30 min per day, or minimum total physical activity of at least 600 MET-minutes/week.

Individuals who did not meet the criteria for HPAL or MPAL were considered to have a low physical activity level (LPAL).

In this study, the HPAL and MPAL groups were combined into the high/medium physical activity level (HMPAL) group and compared with the LPAL group to identify factors associated with individuals with low physical activity. Moderate or higher levels of physical activity in elderly people have been shown to reduce the risk of developing chronic low back pain^18^. IPAQ-SF is a comprehensive, randomized, controlled study of the risk of developing chronic low back pain. In addition, average daily calorie consumption (ADCC) was calculated based on the procedures defined in the IPAQ-SF.

### Chronic pain related measure

Central sensitization inventory-9 (CSI-9) was used to examine central sensitization (CS) in individuals with chronic pain. CS is a physiological phenomenon in which dysregulation of the central nervous system leads to neuronal dysregulation and hyperexcitability, causing hypersensitivity to both noxious and non-noxious stimuli^19^. CSI-9 is a syndromic and self- reported questionnaire consisting of nine items that assess health-related symptoms common to individuals with the CS syndrome associated with CP^20^. CSI-9 is a shortened version of the 25-item CSI, and higher scores indicate more severe CS. Subjects' kinesiophobia was assessed using the Japanese version of the 11-item Tampa scale for kinesiophobia (TSK-11). Kinesiophobia is defined as an excessive, irrational, and debilitating fear of carrying out a physical movement due to a feeling of vulnerability to a painful injury or reinjury^21^. In TSK-11, higher scores indicate greater fear of movement or reinjury^22^.

### Physical function assessment

Grip strength was evaluated as an indicator of the degree of whole-body muscle strength. It was measured using a hand dynamometer (Grip-D; Takei, Niigata, Japan). Participants were instructed to walk 6.4 m (divided into 2-m zones at each end and a 2.4-m zone in the middle) at a speed they found comfortable. The time needed (s) to pass the 2.4-m middle zone was measured to calculate the gait speed (m/s). Participants could use a cane or walker if they were unable to walk without help. The average of five gait trials was used. In addition, a self-administered questionnaire was used to obtain information on history of falls. Subjects responded on whether they had fallen in the past year. Before answering this question, subjects were asked to note the following definition of a history of falls: “A fall is when you lose your balance and some part of your body other than your feet touches the ground. It includes slipping and stumbling. A fall also includes contact with another person, from a bicycle, or from a height, but does not include a fall resulting from a vehicle collision”. This definition was based on Gibson's definition of a fall as: "Unintentionally coming to the ground or some lower level and other than as a consequence of sustaining a violent blow…"^23^.

### Clinical history

Insomnia was assessed using the Athens insomnia scale (AIS)^24^.

### Statistical analysis

Subjects were classified into HMPAL and LPAL groups according to IPAQ-SF classifications. Subjects with missing data were excluded. Unpaired *t* test, Mann Whitney *U* test, and χ^2^ test were used to evaluate significant differences between the HMPAL and LPAL groups. LR was used to identify the factors most strongly related to the physical activity level of individuals with CP. Using LR, the odds ratios (OR) for LPAL were calculated. Two regression models were created, one used crude odds ratio, and the other used adjusted age OR. The sample size required for LR was calculated using power analysis. The minimum sample size required for LR was 87, using a power of 80% and a significance level of 0.05.

Factors most strongly related to the physical activity level of individuals with CP were also extracted using MLP, one of the ANN, and factors with high importance for the independent variables. Due to the small sample size, learning was set at 80%, physical activity level was used as the dependent variable, and all items measured in this study were used as independent variables. The network was set with the minimum and maximum number of units in the hidden layer set to 1 and 50, respectively, and the number of units in the output layer set to 1. The activation function consisted of hyperbolic tangent for both the output layer and the hidden layer. Predictive models with LR and MLP were evaluated by accuracy, area under the curve (AUC), sensitivity, specificity, and F-1 scores. The statistical software used was IBM SPSS Statistics 27 (IBM Corp., Armonk, NY, USA) with a significance level of < 5%.

## Results

### Subject characteristics

**Figure 1:**
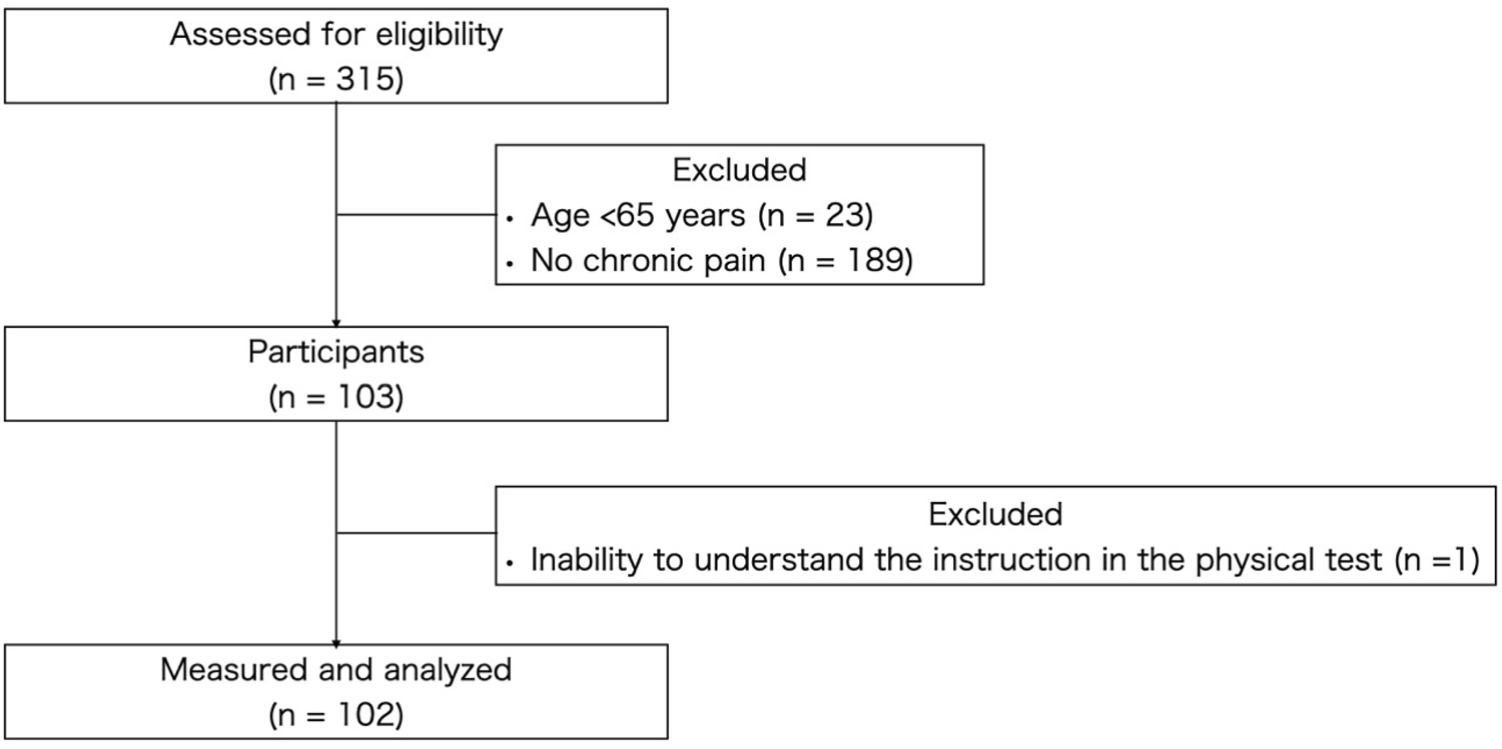
Flowchart illustrating the selection of study participants.

Of the 315 participants who underwent a health check between 2021 and 2022, 103 subjects (mean age, 77.4 ± 5.0 years; males, 24; females, 79), met the inclusion criteria of this study. One subject was excluded because he did not complete the entire study (Fig. 1). According to the IPAQ-SF results, the HMPAL group consisted of 35 subjects and the LPAL group consisted of 67 subjects. The LPAL group had ADCC of 74.0 ± 91.6 kcal, about 10% of the HMPAL group, and the LPAL group more frequently had falls and had a higher BMI than the HMPAL group (Table 1). Interestingly, the subjects in this study did not significantly differ in pain-related outcomes.

**Table 1:** Characteristics of the subjects.

	Total (n = 102)	LPAL (n = 67)	HMPAL (n = 35)	*P* value
ADCC (kcal)	294.6 ± 1376.0	74.0 ± 91.6	717.0 ± 2308.4	
Age (y)	77.4 ± 5.0	75.6 ± 5.9	76.7 ± 6.1	0.42
BMI	22.7 ± 3.2	22.1 ± 3.3	21.8 ± 3.0	0.04
CSI-9	9.6 ± 5.0	9.6 ± 5.0	8.5 ± 5.3	0.31
TSK-11	20.5 ± 6.1	20.8 ± 6.4	20.0 ± 5.0	0.52
History of falls	30	26	4	0.01
Grip strength	21.9 ± 5.9	22.3 ± 6.6	23.4 ± 7.2	0.42
Walking speed (m/s)	1.27 ± 0.25	1.27 ± 0.25	1.27 ± 0.25	0.88
AIS	4.8 ± 3.6	5.1 ± 3.6	3.7 ± 3.2	0.07

Mean ± SD, *ADCC* Average Daily Calorie Consumption, *BMI* Body Mass Index, *CSI-9* The Central Sensitization Inventory-9, *TSK-11* Japanese version of the 11-item Tampa Scale for Kinesiophobia, *AIS* Athens Insomnia Scale.

### Factors associated with LPAL group

CSI-9 and TSK were evaluated as chronic pain-related indices, but univariate analysis results showed no significant differences; a comparison of HMPAL and LPAL showed significant differences in BMI and history of falls. Based on this analysis, history of falls (OR: 4.61) was identified as a factor associated with LPAL in the LR adjusted for age (Table 2).

**Table 2:** Logistic regression analysis identifying the factors associated with the low physical activity level.

Independent variables	Crude OR	95% CI	*P*	aOR	95% CI	*P*
BMI	1.12	0.97–1.30	0.13	1.12	0.97–1.30	0.13
History of falls	4.35	1.35–13.97	0.14	4.61	1.41–15.03	0.01

*OR* Odds Ratio, *95% CI* 95% Confidential Interval, *BMI* Body Mass Index.

The MLP results showed that important predictors of LAPL were age (0.169), grip strength (0.169), history of falls (0.164), CSI-9 (0.131), and AIS (0.126) (Table 3). The assessment of the MLP and LR model in predicting LPAL is shown in Table 4. The assessment of LR and MLP in predicting LPAL was accuracy (81.8% vs 84.6%), AUC (0.75 vs 0.89), sensitivity (0.67 vs 0.87), specificity (0.84 vs 0.78) and F-1 score (0.79 vs 0.87), respectively. The MLP was the best model for predicting hypoactivity in individuals with chronic pain.

**Table 3:** Predictors of low physical activity level by multilayer perceptron analysis.

Factor	Importance
Age	0.169
Grip strength	0.169
History of falls	0.164
CSI-9	0.131
AIS	0.126
BMI	0.080
Walking speed	0.073
TSK-11	0.068
Sex	0.020

*BMI* Body Mass Index, *CSI-9* Central Sensitization Inventory-9, *TSK-11* Japanese version of the 11-item Tampa Scale for Kinesiophobia, *AIS* Athens Insomnia Scale.

**Table 4:** Accuracy of multilayer perceptron and logistic regression prediction model.

	Accuracy (%)	AUC	Sensitivity	Specificity	F-1
LR	81.8	0.75	0.67	0.84	0.79
MLP	84.6	0.89	0.87	0.78	0.87

*LR* Logistic Regression Analysis, *MLP* Multilayer Perceptron, *AUC* Area Under the Curve.

## Discussion

This study attempted to construct a model to predict low physical activity levels with data based on a cross-sectional survey of community-dwelling older adults with CP. Two models, LR and MLP, both found that history of falls predicted low levels of physical activity. Falls among older adults may lead to voluntary activity limitation, social isolation, depression, and adverse effects on quality of life^25^. Studies with a population of individuals with CP have shown that CP in older adults with problems related to physical activity, falls, and kinesiophobia has a significant impact on social participation, level of functioning, and quality of life^26^. Our results support those of previous research. Older adults with CP and low levels of physical activity are either activity-limited due to their history of falls, or their activities are self-inhibited. For older adults with CP and low physical activity levels, special attention should be given to fall prevention to promote physical activity. Older adults with CP are at higher risk for falls^27^. Exercises to improve balance, adjustments to medication, and facilitation of improvement of safety in the home are all important in fall prevention^28^.

MLP found that low level of physical activity in elderly people with CP was extracted from the CSI-9, a pain-related index, in addition to BMI and grip strength. BMI is associated with physical inactivity, and grip strength is a biomarker for falls^29, 30^. CS in relation to CP is associated with hyperalgesia and with greater fatigue^31, 32^. Although it is already known that individuals with CP are negatively affected by CS, the current study found that community-dwelling older adults with CP with low levels of physical activity were more affected by CS than individuals with CP who maintained moderate or high levels of physical activity. It is important for all individuals with CP to maintain or increase to sufficient levels of physical activity, but special attention should be given to individuals with low activity levels of CP. Individuals with CS may be pain sensitive, making it difficult to respond to suggestions to increase physical activity. Community-dwelling older adults with CP with low levels of physical activity may be affected by CS; it may influence an individual's physical activity. Such individuals require additional assessment and treatment for CS.

MLP appeared to have superior predictive model accuracy compared with LR. The AUC of MLP used in this study was 0.89. This is comparable or superior to AUC reported in studies predicting chronic pain and sarcopenia^15, 33^. Advantages of ANN include that they mimic the mechanisms of cerebral neurons, they can identify complex, nonlinear relationships between variables, and that they do not require a specific distribution of data. MLP based on ANN appear to be of clinical value for discriminating inactive community-dwelling elderly people with CP and provide useful information for consideration of appropriate interventions. Many previous studies have used LR when identifying predictors, but in future studies, it may be possible to construct more accurate models by using ANN and machine learning in addition to LR.

A limitation of this study is the small population. It was difficult to collect a sufficient sample of community-dwelling elderly individuals with CP, and this limited the independent variables for constructing predictors of community-dwelling older adults with CP and low activity. However, studies examining factors contributing to low-activity CP have so far compared only healthy subjects. This study is thought to be valuable because it compares highly active CP with low-activity CP. ANN are often used for big data analysis, so further analysis should be continued with more subjects. Studies using ANN for small samples have been published, so we should continue to monitor studies using ANN^34, 35^. This study was able to extract factors that predict low levels of physical activity based on reliable physical activity assessment results in community-dwelling older adults with CP. History of falls is an especially important factor in the low physical activity levels of individuals with CP.

## Conclusion

This study aimed to extract factors that predict low levels of physical activity among community-dwelling elderly people with CP. We used MLP, an ANN, in addition to the conventionally-used LR to predict low levels of activity in CP. Both LR and MLP found that a history of falls was a predictor of low levels of activity. Special attention should therefore be given to fall prevention. In addition, MLP appeared to have superior predictive model accuracy. Constructing more accurate models may be possible by using ANN and machine learning in addition to LR. Our results emphasize the importance of fall prevention in improving the physical activity levels of community-dwelling older adults with chronic pain. Future cross-sectional studies should compare multiple analysis methods to show results with improved accuracy.

## Data Availability

The data that support the findings of this study are available from the corresponding author upon reasonable request.
